# Supplementary material for: Commercial afforestation can deliver effective climate change mitigation under multiple decarbonisation pathways
Source: Nat Commun. 2021 Jun 22;12:3831. doi: 10.1038/s41467-021-24084-x (PMC8219817; doi:10.1038/s41467-021-24084-x)
Supplement: Supplementary file 1 — Supplementary Information [file 41467_2021_24084_MOESM1_ESM.pdf]

Supporting Information for:

Commercial afforestation can deliver effective climate change mitigation under multiple decarbonisation pathways

Eilidh J. Forster[1], John R. Healey[1], Caren Dymond[2] & David Styles[1,3]

[1]School of Natural Sciences, Bangor University, Gwynedd, LL57 2UW; [2]Government of British Columbia, PO BOX 9544, STN PROV GOVT, Victoria BC, V8W9C5, Canada; [3]Bernal Institute, School of Engineering, University of Limerick, Limerick V94 T9PX. Ireland

S1. Elaboration of scenarios and assumptions

The focus of our study is on comparison of long-term greenhouse gas (GHG) emissions and sequestration between typical commercial and conservation forests in the UK, considering the four stages of forestry value chains: production, use, cascading use and end-of life. Each stage is associated with a range of possible activities and emission factors that strongly influence results, and that also depend on wider decarbonisation contexts. To estimate the potentially high uncertainty arising from this, we iteratively designed 33 scenarios that explicitly address key variables, focussing results around seven base case scenarios: four scenarios indicative of different commercial wood uses and decarbonisation contexts, and three main types of conservation forest (Table 1). The remaining 26 scenarios are used to test the sensitivity of results for these seven scenarios to tree growth rate. In this way, the study provides explicit insight into the sensitivity of results to individual mechanisms and their interactions, generating new evidence on linkages between these factors. Mechanisms tested for their influence on climate change mitigation were: forest types and productivity, harvest options, wood products, substituted products, and rate of industrial decarbonisation. Below, we elaborate on our scenario development, indicating how default scenarios underpin robust conclusions because they were derived: (i) from UK national statistics and national decarbonisation pathways proposed by the UK Committee on Climate Change[1]; (ii) with caution, employing the principle of conservatism with respect to headline results.

1.1 Scale

The functional unit of assessment is one hectare of land (aspatial, assuming 'average' soil conditions) so that it can be scaled up to any scale of afforestation plan, such as the UK national strategy of 30,000 ha yr^{-1} until 2050, modelled in this study. CBM-CFS3 is an aspatial carbon model for modelling forest growth and doesn't account for the effects of intimate tree species mixtures or of different harvesting systems (e.g. continuous cover forestry versus clear felling) on species growth rates. Instead, it models the percentages of each species planted to calculate system growth and percentages of carbon harvested to calculate system removals (both factors are user defined). In response to thinning, the default CBM-CFS3 behaviour is for the carbon stock to be reduced, but the future growth increments to be unchanged. So the same growth rate applies to essentially fewer individuals. Hence, although this study does not report the impact of different harvesting rates, the climate change impact of lower harvesting rates could be interpolated from the results for harvested (commercial) and unharvested (conservation) systems.

1.2 Forest types and productivity

Growth rate (or "yield class") differentials are a critical component of the study because carbon sequestration rates and storage in forests are highly dependant upon them[26,27]. Carbon sequestration and harvestable wood available for downstream uses depend on tree growth rate represented by yield class ($m^3ha^{-1}yr^{-1}$), which in turn is highly dependent on tree species and site conditions, including soil type, nutrient availability, temperature, rainfall and wind exposure. *Picea sitchensis* (Sitka spruce) is widely used in commercial forestry in UK, and areas with similar environments in Western Europe, owing to its high yields across a range of environmental conditions[2], and was chosen as the most representative species for commercial forestry in the UK for this study (Table 1). We represent the range of growth rates observed across the majority of commercial Sitka spruce plantations in our analysis by selecting a wide yield class range of 12 to 24 (m^3 ha^{-1} yr^{-1}), based on data from Forest Yield[3] and Mason and Perks (2011)[4]. We take the median of this range, yield class 18, as a conservative default assumption for main results. Future land sparing from agriculture is likely to be on slightly better quality land (e.g. "grade 3b" in the UK Agricultural Land Classification)[5] compared with the marginal (agriculturally unsuitable) land upon which new commercial forests have generally been established in the past. The business case behind commercial forestry operations is based upon realisation of good yields, which could be enhanced in the future via genotype selection in tree breeding programmes. Therefore, we regard our default yield class estimate, based on median existing yields, to be a conservative approach with respect to our conclusions on commercial forests.

Meanwhile, conservation forests are established for different reasons, notably the provision of habitat for conservation-priority species, broader biodiversity conservation, and terrestrial carbon sequestration. However, the native broadleaf tree species favoured in most of the UK for biodiversity purposes generally have slow growth rates, and therefore lower sequestration rates than commercial conifers. We represent these different objectives by evaluating a spectrum of conservation forests, from pure broadleaf forests to fast-growing non-harvested conifer forests. We include the commercial conifer species that are the most abundant in a representative range of forest site types in UK, namely *Picea sitchensis* (Sitka spruce), *Pinus nigra* ssp. *laricio* (Corsican Pine) and *Pseudotsuga menziesii* (Douglas fir). For the native broadleaf species, we included those that are most abundant in forests on the sites most typically used for Sitka spruce forests, namely *Betula pendula* (silver birch), *Sorbus aucuparia* (rowan) and *Quercus robur* (pedunculate oak, but also representative of *Q. petraea*, sessile oak) – see Table 1. In the UK native broadleaf species typically grow much more slowly than the non-native conifers that are used in commercial forestry, especially on low-productivity sites. For native broadleaf species yield class 4 is typical in upland areas on acidic soils where commercial Sitka spruce plantations have been successfully established. Our selection of conservation forest types therefore reflects the full range of conservation forest yield classes, from 4 to 18 (Table 1), with a default assumption of yield class 11 for mixed forests being highly conservative in the context of our conclusions. By including a non-harvested mixed-conifer conservation forest of yield class 18 grown for a carbon sequestration objective, we can analyse the specific effect of harvesting with our results.

In summary, we select a wide range of realistic yield classes for the UK (and similar temperate regions) to ensure a broad comparison of GHG mitigation efficacy between commercial and conservation forests, choosing conservative median values for our main comparisons.

1.3 Forest harvest options

Forest management systems can vary in harvest intensity: 1) No harvesting; 2) Various forms of low impact continuous cover forestry, where trees are harvested frequently but at a low intensity (in small groups or even down to the scale of selected individual trees); and 3) Clear felling, where intensive harvesting of all trees in relatively large blocks is carried out infrequently (once per forest rotation). Clear fell harvesting is often combined with thinning, which is the removal of a percentage of trees mid-rotation to increase spacing between remaining trees and encourage them to grow straighter and more productively. While the key variables are harvest intensity and frequency, the distinction between these forest management systems becomes blurred, with many different kinds of individual silvicultural system that defy simple classification. Therefore, this study assesses the extreme ends of the forest management range, both of which are common in UK: no harvesting versus clear-fell harvesting (with thinning). Clear felling is currently the most common management system for commercial conifer forests in UK and many other temperate countries.

1.4 Use of wood

Wood harvested from forests ends up in a plethora of harvested wood products (HWP), with different durations of carbon storage[6], and potentially substituting for products of differing carbon intensities[7]. We compiled a material flow of wood in the UK from various datasets[8,9] (Fig. 1) to understand the main primary and secondary uses of HWP entering the economy via a sawmill that maximises high quality sawn wood output[10]. This material flow was used to parameterise a hierarchical wood use scenario (Table 1), demonstrating elements of cascading use of wood whilst being representative of typical current UK uses.

An alternative bioenergy-intense wood value chain was also assessed. This scenario provides a contrasting use pathway to the hierarchical scenario in that significantly more energy is produced (instead of materials) leading to lower HWP carbon storage. It also reflects the current prominence of bioenergy and bioenergy carbon capture & storage (BECCS) in global and national climate mitigation scenario modelling[1,11,12]. It is, however, unlikely that high-quality timber would be diverted to typically lower value bioenergy uses. Therefore, material flows in the bioenergy scenario are based on the assumption that high quality sawn wood and associated co-products are still produced, but that 100% of thinnings, 65% of main harvest, and 48% of secondary use flows go into electricity generation (Table 1).

Electricity generation was selected as the primary energy conversion route for wood in both the *Hierarchical* and *Bioenergy* scenarios to reflect the future demand for dispatchable electricity sources, needed to complement an increasing share of intermittent renewable sources (such as wind and solar), and because it is a central component of BECCS targets[1,13]. Energy conversion, energy substitution and deployment of carbon capture & storage (CCS) were modelled dynamically based on decadal progression in line with "*Core*" and "*Further Ambition*" pathways for UK decarbonisation defined by the UK Committee on Climate Change[1] and in other relevant literature[14,15]. These are summarised in Table 2.

More circular use of wood could lead to much longer HWP carbon storage closer to the long-term geological sequestration of biogenic carbon anticipated for BECCS[16]. Substantial research and development is being directed at the use of wood, or constituents of wood such as cellulose, in high performance bio-based materials[17]. Thus our representation of future hierarchical wood use based

on current technology is conservative compared with more circular visions for future wood use proposed in other studies[18].

1.5 Substituted products and energy

Product substitution derives directly from material flows in Fig. 1, and is elaborated in Fig. 2. Substituted products are determined as the marginal product type directly substitutable with the HWP in question[19]. Increased use of sawn wood in construction following forestry expansion is assumed to replace masonry walls, as detailed in the main manuscript. This reflects ambitious UK timber frame house building targets[1] – a shift from the currently more common practice of masonry structural walls. The UK imports 98% of its sawn softwood[20] so the carcassing produced domestically from newly planted forests in this study is assumed to supply this additional timber demand.

Bioenergy replaces *marginal* sources of heat and electricity generation that change on a decadal basis according to decarbonisation pathways[1] as elaborated in Table 2. Notably, natural gas remains the marginal electricity source likely to be substituted by additional dispatchable bioenergy from wood harvested from forests through to 2120 for the *Core* decarbonisation pathway, reflecting the UK Committee of Climate Change projection that CCS is fully deployed later this century. Similarly, although diminishing for electricity generation in the UK, coal remains an indispensable fuel for high temperature furnaces needed in cement production and other heavy industry[14]. Consequently, coal is assumed to remain the main marginal heat source replaced by additional wood fuel through to 2085 (with CCS) in the *Core* context and 2040 in the *Further Ambition* context (Supplementary Data 1). A number of references are cited in Supplementary Data 1 to justify individual assumptions that required more detail than available from projections made by the UK Committee on Climate Change.

We make an important, conservative assumption that considerably reduces uncertainty: deployment of CCS occurs in parallel for fossil fuels and bioenergy. This means that uncertainty over the scale of future CCS deployment is reduced because long-term carbon sequestration via BECCS is inversely related to energy substitution credits as CCS reduces the carbon intensity of substituted fossil energy. This is an important point given the major contribution of BECCS to overall mitigation in the bioenergy scenarios, particularly in the *Further Ambition* context (Fig. 3), and the high uncertainty regarding the commercial viability of BECCS[16,21].

Conservatively, we attribute no substitution credits to HWP where wood is the dominant feedstock, including fence posts, paper, mulch, other short-lived products and boards. Consequently, whilst HWP carbon storage is modelled for these wood uses, we are likely to underestimate substitution credits, especially for the hierarchical scenarios.

1.6 Additional sensitivity analyses

Given the highly complex and uncertain nature of future timber value chains, and in particular future product substitution, we carried out sensitivity analyses on different wood uses and product substitution options (see Supplementary Data 5). For simplicity, the process emissions and HWP carbon storage of alternative wood products tested are assumed to be equal to the base case. Only the impact of alternative product substitutions is considered here.

High quality timber will likely continue to be used predominantly for structural timber (carcassing) in future wood value chains and, in the UK, concrete used in building construction is considered the appropriate *marginal* product substitution for this. However, we note that in some product market contexts (e.g. where there is limited construction of buildings for which timber carcassing is a realistic option, or where concrete is not currently a dominant structural material choice) only a

proportion of the production of this grade of timber will be used to substitute for concrete. Therefore, we added a pessimistic scenario in which only 50% of the carcassing timber substitutes for concrete, with the remaining 50% achieving no product substitution credit.

A significant use of smaller diameter logs in our base case *Hierarchical* value chain is for production of pallets and packaging, which have a relatively short product life and to which we applied no product substitution credit. An alternative use of good quality small diameter timber is for production of engineered timber suitable for high-specification construction uses, such as glued laminated (glulam) beams and cross laminated timber (CLT) panels. Engineered timber is increasingly being considered for mid-rise buildings, and can achieve GHG mitigation via both long term terrestrial carbon storage and avoided emissions from substituted mineral-based structural materials[22]. Sensitivity analysis was performed to test the product substitution effects of diverting small diameter timber from pallets (equating to around 17% of total wood harvested) to the production of glulam to substitute for recycled steel in construction uses. Decarbonisation of steel production was applied, based on an industry roadmap[14].

The dominant use for lower grade timber in the base case *Hierarchical* wood value chain is wood panel production. Wood panels have a medium product life and gain no product substitution credits in our projections. We measure the impact of diverting lower quality timber from wood panel production (equating to around 24% of the total wood harvested) to pulping for the production of viscose fibre, which substitutes for oil-derived polyethylene terephthalate (PET, 'polyester') fibre. Viscose and PET are currently treated as alternatives for many uses in the textiles industry, though PET has advantages in terms of price and some properties such as water repellency[25]. However, the textiles industry is rapidly moving towards more sustainable materials so that, by the time of the first harvest of a newly-planted forest, viscose may occupy a much higher market share. We modelled PET substitution by wood-derived viscose and accounted for possible decarbonisation by applying the same staged decarbonisation rate as that applied in the sensitivity test for structural steel production, given no equivalent decarbonisation roadmap could be found for oil-derived textiles.

Finally, given high uncertainty surrounding the timing of CCS technology deployment, we also tested the sensitivity of results to this. We modelled the unlikely and pessimistic decarbonisation scenario in which there is no deployment of CCS technology to either bioenergy or fossil fuel energy systems (including furnaces used in concrete production) during the study time frame (see Supplementary Data 6-7).

1.7 Example life cycle inventory for hierarchical value chain

Full life cycle inventories for hierarchical and bioenergy value chains are displayed within Supplementary Data 2-3, respectively. Supplementary Table 1 below summarises the main inventory for the hierarchical value chain.

Supplementary Table 1 - Inventory of key inputs and outputs for processes considered along the life cycle of forestry value chains derived from thinned forest systems over 100 years. Emissions factors (EF) and their sources are indicated. GWP is global warming potential (measured in kg CO_2e).

Process stage	Input/output/ process	Activity data source	Units	Thinned In	Thinned Out	EFs GWP	EF source
Site establishment	Land		ha	1			
	15 tonne 360 Excavator	Expert estimate	hr	15		65	Ecoinvent[23]
	Herbicide (glyphosphate)	Industry recommended	kg	1			Ecoinvent[23]
Planting (1&2)	Tree seedlings	GH[8]	Item(s)	50,000		0	Ecoinvent[23]
	15 tonne 360 Excavator	GH[8]	hrs	30		65	Ecoinvent[23]
	Pesticides (acetamiprip)	Industry recommended	kg	2			Ecoinvent[23]
Forest management	Harvester (diesel use)	GH[8]	hrs	78		56	Ecoinvent[23]
	Forwarder (diesel use)	GH[8]	hrs	78		46	Ecoinvent[23]
Forest growth	Net C sequestered	CBM-CFS3[24]	kg C	207,179			IPCC[10]
	Harvested wood	CBM-CFS3[24], GH[8]	m^3		3,701		IPCC[10]
Transport (forest to processor)	>32 t truck, EURO6	GH[8]	t.km	3,089			Ecoinvent[23]
Debarking	Harvested wood	CBM[24], GH[8]	m^3	3,373			
	Diesel	Ecoinvent[23]	MJ	1,690			
	Lubricating oil	Ecoinvent[23]	kg	1			
	Bark chips	GH[8], FR CFs[3]	kg		142,684	20	Ecoinvent[23]
	Debarked wood	GH[8], FR CFs[3]	m^3		3,012		
Sawing	Diesel (internal transport)	Ecoinvent[23]	MJ	37,698			
	Electricity	Ecoinvent[23]	kWh	25,209			
	Lubricating oil	Ecoinvent[23]	kg	137			
	Debarked wood	GH[8], FR CFs[3]	m^3	2491			
	Sawnwood	JJ&S[10]	m^3		1,442	25	Ecoinvent[23]
	Sawmill residues	JJ&S[10]	kg		189,552		
Drying (of sawn timber)	Electricity	Ecoinvent[23]	kWh	24,087			
	Sawnwood	JJ&S[10]	m^3	1,442			
	Sawnwood - dried (u=20%)	Assume no loss in volume during drying	m^3		1,442	29	Ecoinvent[23]
Planing	Electricity	Ecoinvent[23]	kWh	12,506			
	Sawnwood (carcassing) dried (u=20%)	JJ&S[10]	m^3	1,442			
	Sawnwood (carcassing) planed	Vol loss accounted for in 'sawing'	m^3		1,442	35	Ecoinvent[23]
	Sawmill residues	JJ&S[10]	kg		189,552		
Chemical treatment	Electricity	Ecoinvent[23]	kWh	146			
	Wood preservative	Ecoinvent[23]	kg	203,723			
	Sawnwood (fencing) dried (u=20%)	JJ&S[10]	kg	108,539			

Process stage	Input/output/ process	Activity data source	Units	Thinned		EFs	EF source
				In	Out	GWP	
	Debarked wood (fence poles)	GH[8],FR CFs[3]	kg	95,184			
	Preserved wood	No vol. change	kg		203,723	0	Ecoinvent[23]
Particle board production	Electricity	Ecoinvent[23]	kWh	69,770			
	Heat	Ecoinvent[23]	MJ	818,655			
	Resin	Ecoinvent[23]	kg	34,214			
	Debarked wood (chip)	GH[8]	kg	67,991			
	Sawmill residues	JJ&S[10]	kg	170,596			
	Recycled wood	FC report	kg	299,961			
	Particle board	FR CFs[3]	m^3		690	262	Ecoinvent[23]
Fibre board production	Electricity	Ecoinvent[23]	kWh	2			
	Heat	Ecoinvent[23]	MJ	2			
	Debarked wood (chip)	GH[8], FR CFs[3]	kg	22,664			
	Sawmill residues	JJ&S[10]	kg	56,865			
	Fibre board	JJ&S[10], GH[8], FR CFs[3]	m^3		147	98	Ecoinvent[23]
Woodchip production (for biomass energy)	Electricity	Ecoinvent[23]	kWh	3,673			
	Lubricating oil	Ecoinvent[23]	kg	0			
	Harvested wood - 'fuel'	GH[8]	kg	61,715			
	Recycled wood - 'biomass'	FC	kg	80,639			
	Wood chips	GH[8]	kg, dry		142,354	0	Ecoinvent[23]
Biomass energy	Electricity	Ecoinvent[23]	kWh		4,356		Ecoinvent[23]
	Wood chips	GH[8]	Kg, dry	142,354			Conversion biogenic C to CO_2 eq
	Bark chips	GH[8], FR CF[3]	kg	35			
	Sawmill residues	JJ&S	kg	91			
	Heat	Ecoinvent[23]	MJ		783,397	0	Ecoinvent
Graphics paper production	Electricity	Ecoinvent[23]	kWh	29,430			
	Debarked wood - 'pulp'	FR CF[3]	m^3	29			
	Paper, newsprint, virgin	FR CF[3]	kg		10,421	1	Ecoinvent[23]
Graphics paper production (recycled)	Electricity	Ecoinvent[23]	kWh	3,971			
	Recycled paper	GH[8], FR CF[3]	kg	2,482			
	Paper, newsprint, recycled	Mass equal to recycled paper	kg		2,482	1	Ecoinvent[23]
Paperboard production	Electricity	Ecoinvent[23]	kWh	1,013			
	Debarked wood - 'pulp'	GH[8], FR CF[3]	GH	29			
	Board box	GH[8], FR CF[3]	kg		14,452	1	Ecoinvent[23]
HWP in use	C accumulated in HWP	IPCC[10]	kg C	611,725			IPCC[10]
Landfill	Waste wood	FC, Defra[9], IPCC[10]	kg	2, 354			IPCC[10]
	Waste paper	FC, Defra[9], IPCC[10]	kg	644			IPCC[10]
Incineration	Waste wood	FC, Defra[9], IPCC[10]	kg	62,056			Conversion of biogenic C to CO_2eq
	Waste paper	FC, Defra[9], IPCC[10]	kg	0			
	Electricity	Ecoinvent[23]	kWh		176,860		
	Heat	Ecoinvent[23]	MJ		23,962		

Process stage	Input/output/ process	Activity data source	Units	Thinned		EFs	EF source
				In	Out	GWP	
Avoided construction materials	140 mm concrete block and mortar wall replaced by timber frame wall	BRE[25]	m^2	18,779		37	Ecoinvent[23]

References

1. UK CCC. *Net Zero: The UK's contribution to stopping global warming*. https://www.theccc.org.uk/wp-content/uploads/2019/05/Net-Zero-The-UKs-contribution-to-stopping-global-warming.pdf (2019).

2. Waring, R. H. A process model analysis of environmental limitations on the growth of Sitka spruce plantations in Great Britain. *For. An Int. J. For. Res.* **73**, 65–79 (2000).

3. Matthews, R. W., Jenkins, T. A. R., Mackie, E. D. & Dick, E. C. *Forest Yield: A handbook on forest growth and yield tables for British forestry*. https://www.forestresearch.gov.uk/research/forest-yield-a-handbook-on-forest-growth-and-yield-tables-for-british-forestry/ (2016).

4. Mason, B. & Perks, M. P. Sitka spruce (picea sitchensis) forests in Atlantic Europe: Changes in forest management and possible consequences for carbon sequestration. *Scand. J. For. Res.* **26**, 72–81 (2011).

5. Natural England. Agricultural Land Classification: protecting the best and most versatile agricultural land - TIN049. http://publications.naturalengland.org.uk/publication/35012?cache=1591347789.55 (2009).

6. Pawelzik, P. *et al.* Critical aspects in the life cycle assessment (LCA) of bio-based materials – Reviewing methodologies and deriving recommendations. *Resour. Conserv. Recycl.* **73**, 211–228 (2013).

7. Leskinen, P. *et al. Substitution effects of wood-based products in climate change mitigation*. (2018).

8. Gresham House. Forest production data. (2018).

9. DEFRA. ENV23 - UK statistics on waste - GOV.UK. https://www.gov.uk/government/statistical-data-sets/env23-uk-waste-data-and-management (2018).

10. James Jones & Sons. Sawmill production data provided from personal correspondence. (2019).

11. IPCC. *Climate Change and Land. An IPCC Special Report on climate change, desertification, land degradation, sustainable land management, food security, and greenhouse gas fluxes in terrestrial ecosystems*. www.ipcc.ch (2019).

12. UK CCC. *Land use: Policies for a Net Zero UK - Committee on Climate Change*. https://www.theccc.org.uk/publication/land-use-policies-for-a-net-zero-uk/ (2020).

13. Huppmann, D. *et al.* IAMC 1.5°C Scenario Explorer and Data hosted by IIASA. (2019).

14. Material Economics. *Industrial Transformation 2050 Pathways to Net-Zero Emissions from EU*

Heavy Industry. (2019).

15. Vandepaer, L., Treyer, K., Mutel, C., Bauer, C. & Amor, B. The integration of long-term marginal electricity supply mixes in the ecoinvent consequential database version 3.4 and examination of modeling choices. *Int. J. Life Cycle Assess.* **24**, 1409–1428 (2019).

16. Muri, H. The role of large - Scale BECCS in the pursuit of the 1.5°C target: An Earth system model perspective. *Environ. Res. Lett.* **13**, (2018).

17. Hamad, W. Y., Miao, C. & Beck, S. Growing the Bioeconomy: Advances in the Development of Applications for Cellulose Filaments and Nanocrystals. *Ind. Biotechnol.* **15**, 133–137 (2019).

18. Brunet-Navarro, P., Jochheim, H., Kroiher, F. & Muys, B. Effect of cascade use on the carbon balance of the German and European wood sectors. *J. Clean. Prod.* **170**, 137–146 (2018).

19. Weidema, B. P. & Schmidt, J. H. Avoiding Allocation in Life Cycle Assessment Revisited. *J. Ind. Ecol.* **14**, 192–195 (2010).

20. Timber Trade Federation. *TTF Statistical Review 2017: Industry facts and figures for the year 2016*. https://ttf.co.uk/download/ttf-statistical-review-2017/ (2017).

21. Tokimatsu, K., Yasuoka, R. & Nishio, M. Global zero emissions scenarios: The role of biomass energy with carbon capture and storage by forested land use. *Appl. Energy* **185**, 1899–1906 (2017).

22. Churkina, G. *et al.* Buildings as a global carbon sink. *Nature Sustainability* vol. 3 269–276 (2020).

23. Wernet, G. *et al.* The ecoinvent database version 3 (part I): overview and methodology. *Int. J. Life Cycle Assess.* **21**, 1218–1230 (2016).

24. Kull, S. J., Northern Forestry Centre (Canada) & Canada. Natural Resources Canada. *Operational-scale carbon budget model of the Canadian forest sector (CBM-CFS3) : version 1.2, user's guide*. (2016).

25. BRE. IMPACT database v5 (accessed via etool LCA software). (2018).

28. Churkina, G. *et al*. Buildings as a global carbon sink. *Nature Sustainability* **3** *269–276* (2020).

29. Timber Trade Federation (UK). *Statistical Review 2017* (2017)
