# Supplementary material for: Commercial afforestation can deliver effective climate change mitigation under multiple decarbonisation pathways
Source: Nat Commun. 2021 Jun 22;12:3831. doi: 10.1038/s41467-021-24084-x (PMC8219817; doi:10.1038/s41467-021-24084-x)
Supplement: Supplementary file 3 — Description of Additional Supplementary Files [file 41467_2021_24084_MOESM3_ESM.pdf]

Description of Additional Supplementary Files

File Name: Supplementary Data 1
Description: Data 1 is an MS Excel file containing information on marginal energy sources and carbon capture & storage (CCS) deployment throughout the study period (2020 to 2120).

File Name: Supplementary Data 2
Description: Data 2 is an MS Excel file containing all inventory activity and emissions data for the default Sitaka spruce yield class 18 commercial forest linked with a Hierarchical value chain, and also for the semi-natural broadleaf forest (includes outputs from CBM-CFS3 model).

File Name: Supplementary Data 3
Description: Data 3 is an MS Excel file containing all inventory activity and emissions data for the default Sitaka spruce yield class 18 commercial forest linked with a Bioenergy value chain, and also for the semi-natural broadleaf forest (includes outputs from CBM-CFS3 model).

File Name: Supplementary Data 4
Description: Data 4 is an MS Excel file containing graphs of cumulative GHG mitigation through time.

File Name: Supplementary Data 5
Description: Data 5 is an MS Excel file containing inventory and results data for alternative substitution factors applied in sensitivity analyses.

File Name: Supplementary Data 6
Description: Data 6 is a revised version of Data 2, containing full inventory and results for commercial forest linked with a Hierarchical value chain, without future CCS deployment (sensitivity analysis around CCS).

File Name: Supplementary Data 7
Description: Data 7 is a revised version of Data 3, containing full inventory and results for commercial forest linked with a Hierarchical value chain, without future CCS deployment (sensitivity analysis around CCS).
