# Supplementary material for: Commercial afforestation can deliver effective climate change mitigation under multiple decarbonisation pathways
Source: Nat Commun. 2021 Jun 22;12:3831. doi: 10.1038/s41467-021-24084-x (PMC8219817; doi:10.1038/s41467-021-24084-x)
Supplement: Supplementary file 11 — Reporting Summary [file 41467_2021_24084_MOESM11_ESM.pdf]

nature research

Corresponding author(s): David Styles
Last updated by author(s): Mar 25, 2021

Reporting Summary

Nature Research wishes to improve the reproducibility of the work that we publish. This form provides structure for consistency and transparency in reporting. For further information on Nature Research policies, see our Editorial Policies and the Editorial Policy Checklist.

Statistics

For all statistical analyses, confirm that the following items are present in the figure legend, table legend, main text, or Methods section.

n/a	Confirmed	
☒	☐	The exact sample size (*n*) for each experimental group/condition, given as a discrete number and unit of measurement
☒	☐	A statement on whether measurements were taken from distinct samples or whether the same sample was measured repeatedly
☒	☐	The statistical test(s) used AND whether they are one- or two-sided<br>*Only common tests should be described solely by name; describe more complex techniques in the Methods section.*
☒	☐	A description of all covariates tested
☒	☐	A description of any assumptions or corrections, such as tests of normality and adjustment for multiple comparisons
☒	☐	A full description of the statistical parameters including central tendency (e.g. means) or other basic estimates (e.g. regression coefficient) AND variation (e.g. standard deviation) or associated estimates of uncertainty (e.g. confidence intervals)
☒	☐	For null hypothesis testing, the test statistic (e.g. *F*, *t*, *r*) with confidence intervals, effect sizes, degrees of freedom and *P* value noted<br>*Give P values as exact values whenever suitable.*
☒	☐	For Bayesian analysis, information on the choice of priors and Markov chain Monte Carlo settings
☒	☐	For hierarchical and complex designs, identification of the appropriate level for tests and full reporting of outcomes
☒	☐	Estimates of effect sizes (e.g. Cohen's *d*, Pearson's *r*), indicating how they were calculated

Our web collection on statistics for biologists contains articles on many of the points above.

Software and code

Policy information about availability of computer code

Data collection	OpenLCA v1.7.4 was used to extract LCA process data from Ecoinvent v.3.5 database
Data analysis	Analyses were undertaken using the CBM-CFS3 forest growth model and MS Excel.

For manuscripts utilizing custom algorithms or software that are central to the research but not yet described in published literature, software must be made available to editors and reviewers. We strongly encourage code deposition in a community repository (e.g. GitHub). See the Nature Research guidelines for submitting code & software for further information.

Data

Policy information about availability of data

All manuscripts must include a data availability statement. This statement should provide the following information, where applicable:

- Accession codes, unique identifiers, or web links for publicly available datasets
- A list of figures that have associated raw data
- A description of any restrictions on data availability

All data used are embedded in MS Excel files used to undertake the analyses, and available as supplementary files to this manuscript: S2 & S3 for Hierarchical and Bioenergy wood use strategies, respectively. Background data were generated using the publicly-available CBM-CFS3 model and extracted from the Ecoinvent v.3.5 database. All subsequent calculations were undertaken using standard MS Excel functions.

Field-specific reporting

Please select the one below that is the best fit for your research. If you are not sure, read the appropriate sections before making your selection.

☐ Life sciences ☐ Behavioural & social sciences ☒ Ecological, evolutionary & environmental sciences

For a reference copy of the document with all sections, see nature.com/documents/nr-reporting-summary-flat.pdf

Ecological, evolutionary & environmental sciences study design

All studies must disclose on these points even when the disclosure is negative.

Study description	The study involved comparison of 100-yr greenhouse gas mitigation of full value chains (forest growth plus all downstream uses of wood, including product substitutions) for commercial and conservation forests in the UK.
Research sample	Commercial and conservation forests were represented by six forest typologies based on UK statistics: commercial Sitka spruce forests, 100% conifer forests and 100% broadleaf semi-natural conservation forests, plus three combinations of conifer and broadleaf species mixes to represent mixed conservation forests. Commercial forest value chains were subdivided into stylised hierarchical (cascading) wood use and bioenergy wood use. The sample was expanded to include the full range of yield performance associated with the main forest types.
Sampling strategy	The sample of forest typologies and value chain types was based upon UK statistical data for forestry and wood use, elaborated in detail in the paper. The objective was to cover the full range of commercial and conservation forest growth rates (the dominant factor influencing results). The different value chains models, within different decarbonisation pathways, were also selected to cover the range of likely uses of wood.
Data collection	Data were collated via mining of literature, UK statiscal datasets (e.g. from DEFRA), from life cycle assessment databases (Ecoinvent v.3.5) and running the CBM-CFS3 forest growth model. Eilidh Forster, lead author, was responsible for data collection.
Timing and spatial scale	The sample represents the range of UK forest types and yields, whilst dynamic LCA modeling was applied for 100 years into the future (i.e. to 2120).
Data exclusions	NA
Reproducibility	The modeling approach has been fully documented in the Methods and supplementary material - in particular, S2 and S3 files contain all data and embedded calculations used to generate average yield results for commercial hierarchical and bioenergy forestry value chains, respectively.
Randomization	NA
Blinding	NA

Did the study involve field work? ☐ Yes ☒ No

Reporting for specific materials, systems and methods

We require information from authors about some types of materials, experimental systems and methods used in many studies. Here, indicate whether each material, system or method listed is relevant to your study. If you are not sure if a list item applies to your research, read the appropriate section before selecting a response.

Materials & experimental systems

n/a	Involved in the study	
☒	☐	Antibodies
☒	☐	Eukaryotic cell lines
☒	☐	Palaeontology and archaeology
☒	☐	Animals and other organisms
☒	☐	Human research participants
☒	☐	Clinical data
☒	☐	Dual use research of concern

Methods

n/a	Involved in the study	
☒	☐	ChIP-seq
☒	☐	Flow cytometry
☒	☐	MRI-based neuroimaging
